# Respostas Fisiológicas à Caminhada Máxima e Submáxima em Pacientes com Doença Arterial Periférica Sintomática

**DOI:** 10.36660/abc.20200156

**Published:** 2021-08-09

**Authors:** Marcel Chehuen, Aluisio Andrade-Lima, Natan Silva, Roberto Miyasato, Rodrigo W. Alves de Souza, Anthony Leicht, Patricia Chakur Brum, Edilamar M. Oliveira, Nelson Wolosker, Claudia Lucia de Moraes Forjaz

**Affiliations:** 1 Universidade de São Paulo Escola de Educação Física e Esportes São PauloSP Brasil Universidade de São Paulo - Escola de Educação Física e Esportes, São Paulo, SP - Brasil; 2 Universidade Federal de Sergipe AracajuSE Brasil Universidade Federal de Sergipe, Aracaju, SE - Brasil; 3 James Cook University TownsvilleQueensland Austrália James Cook University, Townsville, Queensland - Austrália; 4 Hospital Israelita Albert Einstein São PauloSP Brasil Hospital Israelita Albert Einstein, São Paulo, SP - Brasil

**Keywords:** Caminhada, Doença Arterial Periférica, Velocidade da Caminhada, Monitoração Hemodinâmica, Claudicação Intermitente, estresse Oxidativo, Biomarcadores

## Abstract

**Fundamento:**

Embora a caminhada máxima e submáxima sejam recomendadas para pacientes com doença arterial periférica (DAP), a realização desses exercícios pode induzir diferentes respostas fisiológicas.

**Objetivos:**

Comparar os efeitos agudos de caminhada máxima e submáxima na função cardiovascular, a regulação e os processos fisiopatológicos associados pós-exercício em pacientes com DAP sintomática.

**Métodos:**

Trinta pacientes do sexo masculino foram submetidos a 2 sessões: caminhada máxima (protocolo de Gardner) e caminhada submáxima (15 períodos de 2 minutos de caminhada separados por 2 minutos de repouso ereto). Em cada sessão, foram medidos a pressão arterial (PA), a frequência cardíaca (FC), a modulação autonômica cardíaca (variabilidade da FC), os fluxos sanguíneos (FS) do antebraço e da panturrilha, a capacidade vasodilatadora (hiperemia reativa), o óxido nítrico (ON), o estresse oxidativo (a peroxidação lipídica) e a inflamação (quatro marcadores), pré e pós-caminhada. ANOVAs foram empregadas e p < 0,05 foi considerado significativo.

**Resultados:**

A PA sistólica e a PA média diminuíram após a sessão submáxima, mas aumentaram após a sessão máxima (interações, p < 0,001 para ambas). A PA diastólica não foi alterada após a sessão submáxima (p > 0,05), mas aumentou após a caminhada máxima (interação, p < 0,001). A FC, o equilíbrio simpatovagal e os FS aumentaram de forma semelhante após as duas sessões (momento, p < 0,001, p = 0,04 e p < 0,001, respectivamente), enquanto a capacidade vasodilatadora, o ON e o estresse oxidativo permaneceram inalterados (p > 0,05). As moléculas de adesão vascular e intercelular aumentaram de forma semelhante após as sessões de caminhada máxima e submáxima (momento, p = 0,001).

**Conclusões:**

Nos pacientes com a DAP sintomática, a caminhada submáxima, mas não a máxima, reduziu a PA pós-exercício, enquanto a caminhada máxima manteve a sobrecarga cardíaca elevada durante o período de recuperação. Por outro lado, as sessões de caminhada máxima e submáxima aumentaram a FC, o equilíbrio simpatovagal cardíaco e a inflamação pós-exercício de forma semelhante, enquanto não alteraram a biodisponibilidade de ON e o estresse oxidativo pós-exercício.

## Introdução

A doença arterial periférica (DAP) caracteriza-se pelo estreitamento das artérias dos membros inferiores, convencionalmente devido à aterosclerose.^1^^,^^2^{Norgren, 2007, Inter-society consensus for the management of peripheral arterial disease} Pacientes no segundo estágio da doença (pela classificação de Fontaine) apresentam um sintoma conhecido como claudicação intermitente (CI), que é caracterizada pelo aparecimento de dor na parte inferior da perna durante a caminhada que é aliviada com repouso.^1^^,^^2^ Além disso, pacientes com a DAP sintomática podem apresentar valores elevados de pressão arterial (PA),^3^ sobrecarga cardiovascular,^3^^,^^4^ disfunção autonômica cardíaca,^4^ disfunção endotelial, estresse oxidativo exacerbado e inflamação.^5^^-^^7^ Todas estas manifestações fisiológicas contribuem para a progressão da doença e a morbimortalidade cardiovascular.^2^^,^^3^^,^^6^

O treinamento de exercício tem sido considerado o melhor tratamento para pacientes com a CI.^1^^,^^2^ O treinamento regular melhora a capacidade de locomoção, os sintomas de claudicação, a qualidade de vida e a saúde cardiovascular desses pacientes.^1^^,^^8^^,^^9^ Entre as diversas modalidades de treinamento, a caminhada tem sido amplamente recomendada por várias diretrizes.^1^^,^^2^^,^^9^ No entanto, acredita-se que os efeitos crônicos do treinamento resultem da soma das respostas agudas às sessões,^10^ o que reforça a importância da realização de sessões diárias de caminhada para otimizar as adaptações crônicas. Porém, agudamente, cada sessão de caminhada pode transitoriamente aumentar o risco cardiovascular.^10^ De fato, estudos prévios têm relatado que caminhar até sintomas próximos ao máximo da CI aumenta a sobrecarga cardíaca, disfunção endotelial, estresse oxidativo e inflamação,^7^^,^^11^^-^^13^ o que aumenta o risco de isquemia e arritmias em pacientes predispostos.^14^

Nesse sentido, a caminhada máxima pode ter efeitos perigosos pós-exercício em pacientes com a DAP sintomática, e a caminhada submáxima (até dor moderada nas pernas) se apresenta como uma opção potencial para promover menor sobrecarga cardíaca pós-exercício, acompanhada por estresse oxidativo e inflamação moderados. Novakovic et al.,^15^ mostraram que caminhar com dor moderada melhorou vários desfechos nesses pacientes, como a função vascular. Adicionalmente, estudos anteriores testaram um protocolo específico de caminhada submáxima (15 períodos de 2 minutos de caminhada no limiar da dor), relatando que induziu níveis toleráveis de dor nas pernas, bem como estímulos metabólicos e cardiovasculares moderados durante a sua execução,^16^ induziu hipotensão pós-exercício,^17^ e melhorou a capacidade de caminhada e parâmetros cardiovasculares após um período de treinamento regular.^8^

Desta maneira, o objetivo do presente estudo foi comparar, em pacientes com DAP sintomática, os efeitos agudos de exercícios máximos e submáximos de caminhada nas seguintes variáveis pós-exercício: i) a função cardiovascular, avaliada pela PA, a frequência cardíaca (FC) e o duplo-produto (DP); ii) a modulação autonômica cardíaca, avaliada pelos componentes da variabilidade da FC de frequência baixa (FB) e alta (FA) e pela relação FB/FA; iii) a função vascular, avaliada pelos fluxos sanguíneos (FS) do antebraço e da panturrilha e pelas respostas dos FS à hiperemia reativa; iv) a função endotelial, avaliada pela biodisponibilidade de óxido nítrico (ON); v) o estresse oxidativo, avaliado pela peroxidação lipídica; e vi) a inflamação, avaliada pela proteína C-reativa (CRP), o fator de necrose tumoral-α (TNF-α), a molécula de adesão de células vasculares (VCAM) e a molécula de adesão intercelular (ICAM). As hipóteses eram as seguintes: i) a sessão máxima de caminhada aumentaria a sobrecarga cardíaca pós-exercício (PA, FC e DP), o equilíbrio simpatovagal (FB e relação FB/FA) e a disfunção vascular, enquanto a sessão de caminhada submáxima diminuiria a PA e o DP, enquanto induziria um aumento menor da FC e do equilíbrio simpatovagal; e ii) as sessões máximas e submáximas de caminhada aumentariam o estresse oxidativo pós-exercício e a inflamação com maiores respostas após a caminhada máxima.

## Métodos

Este estudo unicêntrico seguiu um desenho de medição repetida não aleatória em que cada paciente foi submetido a duas sessões experimentais conduzidas em uma ordem fixa. O protocolo do estudo estava de acordo com a Declaração de Helsinque. Foi registrado no site Brasileiro de Ensaios Clínicos (http://www.ensaiosclinicos.gov.br, RBR-3pq58k) e aprovado pelo Comitê Conjunto de Ética em Pesquisa em Seres Humanos da Escola de Educação Física e Esporte da Universidade de São Paulo (processo 667.382). Foi obtido o consentimento informado por escrito de todos os participantes.

### Participantes

Os pacientes foram recrutados entre os atendidos na Unidade Vascular do Hospital das Clínicas da Universidade de São Paulo, Brasil, de acordo com a possibilidade de entrar em contato com eles. Foram convidados pacientes do sexo masculino com diagnóstico prévio de DAP e CI. Os critérios de inclusão foram os seguintes: a) idade ≥ 50 anos; b) índice tornozelo-braquial ≤ 0,90 em pelo menos uma perna^1^; c) estágio II (a e b) de DAP pela classificação de Fontaine^1^; d) índice de massa corporal < kg/m^2^; e) PA sistólica de repouso < 160 mmHg e PA diastólica de repouso < 105 mmHg; f) não uso de β-bloqueadores ou bloqueadores dos canais de cálcio não diidropiridínicos; g) a capacidade de caminhar durante pelo menos 2 minutos a 3,2 km/h em esteira; h) a capacidade de realizar um teste incremental em esteira limitada por sintomas da CI; e i) a ausência de isquemia miocárdica ou arritmias complexas durante teste em esteira.

### Avaliações preliminares

Todos os pacientes foram submetidos a uma avaliação preliminar para identificar se atendiam aos critérios do estudo. Foram entrevistados para avaliar o seguinte: idade, presença de doença cardiovascular, fatores de risco, comorbidades e medicação atual. O índice tornozelo-braquial foi medido conforme descrito anteriormente.^1^ A massa corporal e a estatura foram avaliadas com equipamento padrão (Welmy 110, Brasil) e foi calculado o índice de massa corporal. Foi medida a PA braquial em repouso pelo método auscultatório após 5 minutos de repouso sentado. Três medições foram realizadas em cada uma das 2 visitas e foi calculado o valor médio para cada braço. Também foi documentado o maior valor médio. Por fim, todos os pacientes realizaram um teste ergométrico em esteira seguindo o protocolo de Gardner (3,2 km/h com aumento de 2% no grau por minuto)^18^ até que ocorresse dor máxima de claudicação. Este teste também foi empregado como uma familiarização ao esforço máximo.

### Protocolo experimental

Após os procedimentos preliminares, os pacientes que preencheram todos os critérios do estudo foram submetidos ao protocolo experimental que consistia em ambas as sessões experimentais, caminhada máxima e submáxima. A sessão de caminhada submáxima foi realizada após a sessão máxima com intervalo de pelo menos 7 dias entre as sessões. Além disso, todos os pacientes realizaram 2 sessões de familiarização antes de serem submetidos à sessão submáxima. Para cada sessão, foram avaliadas as variáveis cardiovasculares, autonômicas, endoteliais, de estresse oxidativo e inflamatórias antes e depois dos protocolos de caminhada submáxima ou máxima.

Antes de ambas as sessões, os pacientes foram orientados a manter rotinas semelhantes durante as 24 horas anteriores. Adicionalmente, foram orientados a evitar exercício físico durantes as 48 horas anteriores, bebidas alcoólicas durante as 24 horas anteriores e uso de tabaco no dia das sessões. Também foram orientados a tomar a medicação regularmente e a comparecer ao laboratório em estado de jejum.

As sessões foram realizadas em laboratório com temperatura controlada (20 a 22 °C). Os pacientes chegaram às 7 horas e receberam uma refeição padronizada (2 barras de cereais e 50 ml de suco).^19^^,^^20^ Em seguida, um cateter foi inserido na veia antecubital do braço esquerdo e mantido patente por solução salina estéril. Os pacientes então permaneceram em posição supina durante 20 minutos até o início dos procedimentos experimentais.

Foram iniciados os procedimentos experimentais às 8 horas com avaliações pré-exercício realizadas na posição supina após um período de estabilização de 10 minutos. Eletrocardiograma (ECG) e respiração foram registrados entre 10 e 20 minutos para avaliar a modulação autonômica cardíaca. Foram medidas a PA e a FC auscultatórias em triplicata entre 20 e 25 minutos e foi utilizado o valor médio para análise. Subsequentemente, foi coletada uma amostra de sangue venoso, seguida da avaliação dos FS dos membros inferiores e superiores e das respostas vasodilatadoras à hiperemia reativa.

Na sequência, os pacientes realizaram o exercício de caminhada em esteira. Na sessão máxima, caminharam a 3,2 km/h com o aumento de grau de 2% a cada minuto até a dor máxima (protocolo de Gardner).^18^ Durante a sessão submáxima, realizaram 15 períodos de 2 minutos de caminhada separados por 2 minutos de repouso ereto, conforme descrito anteriormente.^8^^,^^16^^,^^17^ A velocidade da esteira foi mantida em 3,2 km/h com o grau ajustado para manter a FC do limiar de dor (ou seja, a FC medida quando os pacientes experimentaram dor claudicante inicial durante o teste de caminhada máxima preliminar).

Ao final das sessões de caminhada, os pacientes retornaram imediatamente à posição supina para as avaliações pós-exercício que incluíram uma amostra imediata de sangue. Dos 20 aos 30 minutos de recuperação, foram registrados o ECG e os movimentos respiratórios para avaliação da modulação autonômica cardíaca, seguida pelas avaliações da PA e FC auscultatórias em triplicata. Finalmente, foram registrados os FS e as respostas vasodilatadoras.

### Medidas

#### Função cardiovascular

Foram obtidos os registros do ECG no D2 (EMG System, Brasil) com a FC determinada pelo ECG. Foi obtido o sinal respiratório por uma cinta piezoelétrica (UFI, Pneumotrace2, EUA) posicionado no tórax dos pacientes. Foi medida a PA auscultatória no braço dominante utilizando um esfigmomanômetro de mercúrio (Unitec, Brasil), e foi calculada a PA média. O DP foi calculado pelo produto da FC e da PA sistólica como um marcador do consumo de oxigênio miocárdico e, portanto, da sobrecarga cardíaca.^21^

#### Modulação autonômica cardíaca

Para a avaliação autonômica cardíaca, os intervalos R-R do ECG e os sinais respiratórios da cinta torácica foram inseridos em um sistema de aquisição de dados (WinDaq, DI-720, Akron, EUA), com uma taxa de amostragem de 500 Hz/canal. Foram analisados segmentos estacionários de 250 a 300 batimentos por meio de análise espectral da variabilidade da FC usando o método autoregressivo (Heart Scope, versão 1.3.0.1, AMPS-LLC, EUA). Os componentes da variabilidade da FC de FB (FB_RR_, 0,04 – 0,15 Hz) e FA (FA_RR_, 0,15 – 0,4 Hz) foram calculados e expressos em unidades normalizadas (un). Também foi calculada a relação FB/FA. Todos os procedimentos seguiram a *Task Force for HR variability*.^22^

#### Função vascular

Foram determinados os FS simultaneamente no antebraço dominante e na perna com o menor índice tornozelo-braquial, via pletismografia de oclusão venosa (Hokanson, AI6, EUA).^23^ Resumidamente, os FS da mão e do pé foram interrompidos por manguitos insuflados a 200 mmHg posicionados, respectivamente, ao redor do punho e tornozelo. Outros manguitos colocados no braço e na coxa foram insuflados rapidamente por 10 segundos a 40 a 60 mmHg, seguidos por 10 segundos de desinsuflação. Foram detectados aumentos nos volumes do antebraço e da panturrilha por medidores de pressão de mercúrio, posicionados na maior circunferência desses segmentos dos membros e registrados por software especializado (NIVP3; Hokanson, EUA). Foram realizadas as medições durante 4 minutos (12 ciclos de 20 segundos) e as primeiras 2 e a última medição do ciclo foram excluídas da análise (ou seja, uma média de 9 ciclos). As respostas vasodilatadoras do antebraço e da panturrilha à hiperemia reativa foram avaliadas imediatamente após a determinação dos FS.^23^ Para isso, foi ocluído o FS para cada membro durante 5 minutos com a insuflação dos manguitos da coxa e do antebraço a 200 mmHg. Em seguida, os manguitos foram desinsuflados e foram medidos os FS pós-oclusão durante 4 minutos, conforme descrito anteriormente. A resposta vasodilatadora foi calculada como a diferença na área sob a curva (AUCFS) das medições de FS pós e pré-hiperemia

#### Análise de sangue

Em cada momento de amostragem, foram coletados 15 ml de sangue em tubos vacutainer tratados com EDTA anticoagulante de padrão. As amostras foram centrifugadas em até 30 minutos, divididas em alíquotas e armazenadas a –80 °C até a análise. Foram determinadas as concentrações plasmáticas de CRP, TNF-α, VCAM e ICAM por ensaios imunoenzimáticos (ELISA) de acordo com as instruções do fabricante de cada kit (Cayman Chemical, EUA para CRP; and R&D Systems, EUA para TNF-α, VCAM e ICAM). A peroxidação lipídica foi analisada por kits específicos (Cayman Chemical, EUA), e o ON foi analisado pelo método de quimioluminescência com um analisador específico (Sievers ® Nitric Oxide Analyzer ONA 280, EUA).

### Análises estatísticas

Considerando um poder de 90%, um erro alfa de 5% e um desvio padrão de 3 mmHg para a PA sistólica e 0,6 ml.100 ml tecido^−1^.min^−1^ a para BF (ou seja, os desfechos clínicos principais), os tamanhos mínimos de amostra necessários para detectar uma diferença de 4 mmHg na PA sistólica e de 0,5 ml.100 ml tecido^−1^.min^−1^ na BF foram calculados em 10 e 14 indivíduos, respectivamente. Tendo em vista que foram incluídas outras variáveis com maior variação no estudo, o tamanho de amostra utilizado foi maior.

Foram verificadas a normalidade e a homogeneidade de variância para todos os dados pelos testes de Shapiro-Wilk e Levene, respectivamente. Quando identificada a não normalidade dos dados, foi aplicada uma transformação logarítmica e foi obtida a distribuição normal. As respostas às sessões de caminhada foram comparadas por ANOVA bidirecional (Statsoft, Statistic for Windows 4.3, Oklahoma, EUA) para medidas repetidas com a sessão (máxima versus submáxima) e o momento (pré versus pós-exercício) como os fatores principais. Quando os valores pré-exercício foram significativamente diferentes entre as sessões (para FC e DP), empregou-se uma análise de covariância (ANCOVA) usando o valor pré-exercício como covariável. Foi utilizado o teste post-hoc de Newman-Keuls para identificar as significâncias quando apropriado. Foi considerado significativo p < 0,05 e os dados foram apresentados como média ± desvio padrão (DP) para variáveis contínuas e como frequência de aparecimento (%) para variáveis categóricas, como comorbidades e uso de medicamentos.

## Resultados

Inicialmente, 50 pacientes se ofereceram como voluntários para o estudo, 11 dos quais se abstiveram de participar devido à falta de tempo. Deste modo, 39 pacientes assinaram o termo de consentimento livre e esclarecido e realizaram os exames preliminares, dos quais 9 foram excluídos (5 devido a anormalidades de ECG no teste ergométrico e 4 devido à interrupção do teste ergométrico por outros motivos que não a dor claudicante). Portanto, 30 pacientes realizaram as sessões experimentais máximas e submáximas e suas características são apresentadas na Tabela 1.

**Tabela 1:** Características dos pacientes

	Média ± desvio padrão
Idade (anos)	66 ± 11
Índice de massa corporal (kg/m^2^)	25,3 ± 3,2
**Diagnóstico da DAP**	
ITB de repouso	0,62 ± 0,12
DIC (m)	218 ± 87
DTC (m)	606 ± 275
**Comorbidades**	
Obesidade (%)	10,0
Hipertensão (%)	73,3
Diabetes mellitus (%)	26,7
Dislipidemia (%)	93,3
Tabagismo (%)	33,3
Doença cardíaca/acidente vascular cerebral (%)	23,3
**Terapia farmacológica**	
Aspirina (%)	93,3
Estatina (%)	93,3
Agente antihipertensivo (%)	60,0
Hipoglicêmico oral (%)	26,7

Os dados são média ± desvio padrão ou porcentagem (%). DAP: doença arterial periférica; DIC: distância do início da claudicação; DTC: distância total da caminhada; ITB: índice tornozelo-braquial. Obesidade definida como índice de massa corporal > 30 kg/m^2^. Diabetes, hipertensão, dislipidemia, doenças cardíacas e acidente vascular cerebral definidos por diagnóstico médico prévio.

As respostas hemodinâmicas e autonômicas são apresentadas na Tabela 2. A PA sistólica e média diminuíram após a sessão submáxima e aumentaram após a sessão máxima (interações, p < 0,001 para ambas). A PA diastólica aumentou apenas após a caminhada máxima (interação, p < 0,001). A FC e o DP pré-exercício foram significativamente maiores na sessão submáxima de caminhada do que na máxima, e a ANCOVA revelou que estas diferenças pré-exercício não afetaram os resultados. Assim, a FC apresentou aumentos semelhantes após os períodos de caminhada máxima e submáxima (momento, p < 0,001), enquanto o DP aumentou significativamente apenas após a caminhada máxima (interação, p = 0,007).

**Tabela 2:** Variáveis hemodinâmicas e autonômicas medidas pré e pós-exercício nas sessões de caminhada submáxima e máxima

	Submáxima		Máxima				
	Pré	Pós	Pré	Pós	p sessão	p momento	p interação
**Hemodinâmica sistêmica (N = 30)**							
PA sistólica (mmHg)	132 ± 16	125 ± 15*#	134 ± 13	138 ± 17*	0,01	0,18	0,01
PA diastólica (mmHg)	77 ± 9	76 ± 8#	78 ± 8	83 ± 9*	0,01	0,01	0,01
PA média (mmHg)	95 ± 10	92 ± 9*#	96 ± 9	101 ± 10*	0,01	0,05	0,01
FC (bpm)	64 ± 9#	67 ± 9*#	68 ± 9	71 ± 10*	0,01	0,01	0,70
DP (bpm* mmHg)	8466 ± 1466#	8308 ± 1433#	9010 ± 1394	9762 ± 1671*	0,01	0,01	0,01
**Modulação autonômica (n=22)**							
FB (un)	56 ± 22	64 ± 20*	51 ± 18	60 ± 21*	0,12	0,05	0,75
FA (un)	40 ± 21	31 ± 19*	44 ± 17	34 ± 19*	0,19	0,02	0,88
Relação FB/FA	0,2 ± 0,5	0,4 ± 0,4*	0,1 ± 0,4	0,3 ± 0,5*	0,11	0,04	0,74
**Hemodinâmica local (n = 21)**							
FS do antebraço	1,42 ± 0,63	1,68 ± 0,68*	1,41 ± 0,59	1,65 ± 0,67*	0,70	0,01	0,59
FS da panturrilha	12,0 ± 7,1	13,7 ± 7,0*	12,2 ± 7,8	14,4 ± 8,8*	0,11	0,01	0,57
RV do antebraço	80,9 ± 34,8	67,2 ± 30,0*	83,9 ± 40,6	73,6 ± 34,1*	0,18	0,01	0,14
RV da panturrilha	56,8 ± 30,2	40,4 ± 19,8*	63,9 ± 29,5	52,4 ± 26,5*	0,02	0,01	0,52
AUCFS do antebraço	1085 ± 507	1299 ± 609	1294 ± 676	1218 ± 476	0,66	0,38	0,50
AUCFS da panturrilha	1081 ± 606	1152 ± 603	999 ± 467	1270 ± 825	0,89	0,13	0,20

Os dados são média ± desvio padrão. AUC: área sob a curva; DP: duplo-produto; FA: frequência alta; FB: frequência baixa; FC: frequência cardíaca; FS: fluxo sanguíneo; PA: pressão arterial; RV: resistência vascular; un: unidades normalizadas. Valores para FS são ml.100 ml tecido^−1^.min^−1^.

* diferente do pré na mesma sessão (p < 0,05)

# diferente da sessão máxima no mesmo momento (p < 0,05). Análises realizadas por ANOVA bilateral.

A FA diminuiu enquanto a FB e a relação FB/FA aumentaram significativamente e de forma semelhante após as sessões de caminhada máxima e submáxima (momento, p = 0,02, p = 0,05 e p = 0,04, respectivamente).

Os FS do antebraço e da panturrilha aumentaram significativamente e de forma semelhante após as sessões de caminhada máxima e submáxima (momento, p < 0,001), enquanto a resistência vascular do antebraço e da panturrilha diminuiu de forma semelhante após ambos os períodos de caminhada (momento, p < 0,001 e p = 0,01, respectivamente), e a AUCFS do antebraço e da panturrilha não se alterou após os períodos de caminhada submáxima ou máxima (todos p > 0,05).

As respostas sanguíneas são mostradas na Tabela 3. ON, peroxidação lipídica, CRP e TNF-α não se alteraram após as sessões de caminhada submáxima ou máxima (todos p > 0,05), enquanto ICAM e VCAM exibiram um aumento semelhante e significativo após as sessões de caminhada máxima e submáxima (momento, p = 0,001 para ambos).

**Tabela 3:** Concentrações plasmáticas de óxido nítrico, estresse oxidativo e variáveis inflamatórias medidas pré e pós-exercício nas sessões de caminhada submáxima e máxima

	Submáxima		Máxima				
	Pré	Pós	Pré	Pós	p sessão	p momento	p interação
ON (μM)	14,32±5,65	13,59±4,63	13,53±4,51	13,68±4,21	0,29	0,24	0,57
**Estresse oxidativo**							
PL (μM)	18,81±14,69	19,29±15,34	18,71±17,06	20,55±19,01	0,81	0,44	0,77
**Inflamação**							
CRP (pg/ml)	1868±1435	1843±1485	1614±1651	1837±1586	0,41	0,13	0,45
TNF-α (pg/ml)	1,18±0,36	1,24±0,29	1,21±0,28	1,23±0,25	0,75	0,21	0,57
ICAM (ng/ml)	223±96	236±99*	218±92	244±100*	0,74	0,01	0,08
VCAM (ng/ml)	619±250	671±286*	592±237	650±247*	0,16	0,01	0,75

Os dados são média ± desvio padrão. CRP: proteína C-reativa; ICAM: molécula de adesão intercelular; ON: óxido nítrico; PL: peroxidação lipídica; TNF-α: fator de necrose tumoral-α; VCAM: molécula de adesão de células vasculares.

* diferente do pré na mesma sessão (p < 0,05). Análises realizadas por ANOVA bilateral.

## Discussão

Os achados principais do presente estudo foram que os pacientes com a DAP sintomática apresentaram: 1) redução da PA sistólica após a caminhada submáxima, bem como aumento da PA sistólica após a caminhada máxima; 2) aumento do DP apenas após a caminhada máxima; 3) aumentos semelhantes nos níveis de FC, relação FB/FA, FB, ICAM e VCAM após as sessões de caminhada máxima e submáxima; e 4) nenhuma alteração no ON ou na capacidade vasodilatadora após as sessões de caminhada máxima ou submáxima.

Realizar caminhada até a dor submáxima, mas não máxima, diminuiu a PA pós-exercício. Estudos anteriores^17^^,^^24^ já relataram a ocorrência da hipotensão pós-exercício (HPE, ou seja, uma diminuição da PA após uma sessão de exercício em comparação com os valores pré-exercício)^25^^,^^26^ em pacientes com DAP sintomática após realizar caminhada até dor moderada. A novidade deste estudo foi fornecer evidências de que, em pacientes com DAP no estágio II de Fontaine, a HPE não ocorreu quando a caminhada foi realizada até a dor máxima e a PA permaneceu elevada após a caminhada máxima. Visto que a HPE é conhecida como um fenômeno clinicamente relevante em populações hipertensas,^27^ a caminhada submáxima, mas não máxima, pode produzir benefícios hipotensivos agudos em pacientes com CI e hipertensão. Além disso, evidências recentes têm mostrado que a HPE se correlaciona com diminuições na PA após um período de treinamento e é um possível preditor da responsividade crônica.^28^^,^^29^ Desta maneira, estes resultados levantam a hipótese de que a caminhada submáxima pode produzir melhores efeitos hipotensivos crônicos do que a caminhada máxima nesta população. Isto precisa ser testado em estudos futuros.

A FC pós-exercício aumentou de forma semelhante após as sessões de caminhada máxima e submáxima, o que é consistente com o aumento semelhante observado nas alterações da modulação autonômica cardíaca em direção à predominância simpática após ambas as sessões de caminhada (ou seja, um aumento semelhante na FB e na relação FB/FA, bem como uma diminuição na FA).^30^ Esta ausência de diferença entre as sessões máximas e submáximas foi, em certa medida, inesperada, visto que, em outras populações, as alterações na FC pós-exercício e na modulação simpatovagal costumam estar associadas à intensidade do exercício.^31^ Este resultado aparentemente contraditório pode ser explicado pelo fato de a sessão de caminhada submáxima ter durado mais (30 minutos, distância total percorrida = 1600 m) do que a sessão máxima (12 ± 5 minutos, distância total percorrida = 606 ± 275 m). Assim, como a dor produz ativação simpática,^32^ é possível que, apesar da intensidade moderada, o maior período de dor na sessão submáxima possa ter levado a um aumento sustentado da modulação simpática e, consequentemente, da FC durante o período de recuperação, igualando-se ao aumento produzido pela sessão máxima mais intensa, mas mais curta. Adicionalmente, embora a FC pós-exercício tenha aumentado em ambas as sessões de caminhada, a PA diminuiu apenas na sessão de caminhada submáxima, consequentemente levando a um DP mais elevado após a caminhada máxima, o que reflete maior sobrecarga cardíaca e, consequentemente, maior risco de eventos adversos agudos após a caminhada máxima.^14^ Portanto, estes resultados sugerem que a caminhada submáxima pode ser mais segura para pacientes predispostos a eventos cardiovasculares agudos.

Os FS do antebraço e da panturrilha aumentaram de forma semelhante após as sessões de caminhada submáxima e máxima, e essas respostas estão de acordo com estudos anteriores.^17^^,^^33^ No entanto, é de interesse notar que a capacidade vasodilatadora não alterou após qualquer uma das duas sessões de caminhada, embora estudos anteriores tenham relatado uma diminuição da função endotelial após caminhada máxima.^12^^,^^34^ Possíveis diferenças entre os estudos podem estar relacionadas aos métodos usados para avaliar a função vascular (pletismógrafo versus ultrassom). No entanto, no presente estudo, a ausência de alterações na capacidade vasodilatadora está de acordo com a manutenção dos marcadores do ON e do estresse oxidativo.

Conforme o esperado, as sessões de caminhada máxima e submáxima aumentaram os marcadores inflamatórios. Porém, diferentemente da hipótese, a inflamação aumentou de forma semelhante após as duas sessões. Mais uma vez, essa resposta pode estar relacionada ao fato de que a duração do exercício foi maior na sessão de caminhada submáxima, levando a uma magnitude semelhante de inflamação, apesar do menor grau da dor.

A ausência de volume pareado entre as duas sessões de caminhada é uma limitação deste estudo, o que nos impede de atribuir os resultados apenas ao grau de dor. No entanto, como um estudo inicial a comparar as respostas máximas e submáximas pós-exercício, o presente estudo optou pela utilização de um protocolo máximo amplamente investigado na literatura^7^^,^^11^^,^^34^ e um protocolo submáximo, ambos os quais já demonstraram produzir benefícios cardiovasculares.^8^^,^^17^ Estudos futuros devem comparar outros protocolos máximos e submáximos com volume semelhante. Além disso, é importante mencionar que este estudo foi realizado com homens nos estágios IIa e IIb de Fountain, e as respostas pós-caminhada podem diferir em mulheres, em pacientes em outros estágios da doença e em pacientes com características clínicas diferentes, apesar de estágio II de Fontaine. Estudos futuros poderão superar essas limitações estudando mulheres e outros pacientes com DAP. Além disso, as medidas foram realizadas em apenas um momento durante o período pós-exercício. Para melhor compreensão das respostas, deve ser realizado um acompanhamento durante um período mais longo, com mais medidas, em investigações futuras.

## Conclusões

Em pacientes do sexo masculino com a DAP sintomática, caminhar até a dor submáxima, mas não máxima, reduziu a PA pós-exercício, enquanto apenas a caminhada máxima elevou o DP pós-exercício. Por outro lado, as sessões de caminhada máxima e submáxima produziram aumentos pós-exercício semelhantes na FC, equilíbrio simpatovagal cardíaco, FS e inflamação.

### Implicações práticas

- Caminhada submáxima, mas não máxima, reduz a PA no período pós-exercício.
- Apenas a caminhada máxima aumenta a carga cardíaca pós-exercício.
- Caminhada submáxima e máxima aumentam a inflamação pós-exercício de modo semelhante.
- A caminhada submáxima pode ser mais adequada do que a caminhada máxima para pacientes com DAP sintomática, porque resulta em menor risco cardiovascular agudo durante o período de recuperação.
