# Supplementary material for: Respostas Fisiológicas à Caminhada Máxima e Submáxima em Pacientes com Doença Arterial Periférica Sintomática
Source: Arq Bras Cardiol. 2021 Aug 9;117(2):309–16. [Article in Portuguese] doi: 10.36660/abc.20200156 (PMC8395785; doi:10.36660/abc.20200156)
Supplement: Translation

# Translation: Physiological Responses to Maximal and Submaximal Walking in Patients with Symptomatic Peripheral Artery Disease

**Authors:** Marcel Chehuen, Aluisio Andrade-Lima, Natan Silva, Roberto Miyasato, Rodrigo W. Alves de Souza, Anthony Leicht, Patricia Chakur Brum, Edilamar M. Oliveira, Nelson Wolosker, Claudia Lucia de Moraes Forjaz

**Affiliations:** 1 São PauloSP Brazil Universidade de São Paulo - Escola de Educação Física e Esportes, São Paulo, SP - Brazil; 2 AracajuSE Brazil Universidade Federal de Sergipe, Aracaju, SE - Brazil; 3 TownsvilleQueensland Australia James Cook University, Townsville, Queensland - Australia; 4 São PauloSP Brazil Hospital Israelita Albert Einstein, São Paulo, SP - Brazil

**Keywords:** Walking, Peripheral Arterial Disease, Walking Speed, Hemodynamic Monitoring, Intermittent Claudication, Oxidative Stress, Byomarkers

## Abstract

**Background:**

Although maximal and submaximal walking are recommended for patients with peripheral artery disease (PAD), performing these exercises may induce different physiological responses.

**Objectives:**

To compare the acute effects of maximal and submaximal walking on post-exercise cardiovascular function, regulation, and associated pathophysiological processes in patients with symptomatic PAD.

**Methods:**

Thirty male patients underwent 2 sessions: maximal walking (Gardner's protocol) and submaximal walking (15 bouts of 2 minutes of walking separated by 2 minutes of upright rest). In each session, blood pressure (BP), heart rate (HR), cardiac autonomic modulation (HR variability), forearm and calf blood flows (BF), vasodilatory capacity (reactive hyperemia), nitric oxide (NO), oxidative stress (lipid peroxidation), and inflammation (four markers) were measured pre- and post-walking. ANOVAs were employed, and p < 0.05 was considered significant.

**Results:**

Systolic and mean BP decreased after the submaximal session, but they increased after the maximal session (interactions, p < 0.001 for both). Diastolic BP did not change after the submaximal session (p > 0.05), and it increased after maximal walking (interaction, p < 0.001). HR, sympathovagal balance, and BF increased similarly after both sessions (moment, p < 0.001, p = 0.04, and p < 0.001, respectively), while vasodilatory capacity, NO, and oxidative stress remained unchanged (p > 0.05). Vascular and intercellular adhesion molecules increased similarly after both maximal and submaximal walking sessions (moment, p = 0.001).

**Conclusions:**

In patients with symptomatic PAD, submaximal, but not maximal walking reduced post-exercise BP, while maximal walking maintained elevated cardiac overload during the recovery period. On the other hand, maximal and submaximal walking sessions similarly increased post-exercise HR, cardiac sympathovagal balance, and inflammation, while they did not change post-exercise NO bioavailability and oxidative stress.

## Introduction

Peripheral artery disease (PAD) is characterized by the narrowing of the lower limb arteries, conventionally due to atherosclerosis.^1^^,^^2^{Norgren, 2007, Inter-society consensus for the management of peripheral arterial disease} Patients at the second stage of the disease (Fontaine classification) present a symptom known as intermittent claudication (IC), which is characterized by the appearance of pain in the lower leg during walking that is relieved with rest.^1^^,^^2^ Additionally, patients with symptomatic PAD could present high blood pressure (BP) values,^3^ cardiovascular overload,^3^^,^^4^ cardiac autonomic dysfunction,^4^ endothelial dysfunction, exacerbated oxidative stress, and inflammation.^5^^-^^7^ All these physiological manifestations contribute to the progression of the disease and cardiovascular morbimortality.^2^^,^^3^^,^^6^

Exercise training has been considered the best treatment for patients with IC.^1^^,^^2^ Regular training improves these patients’ walking capacity, claudication symptoms, quality of life, and cardiovascular health.^1^^,^^8^^,^^9^ Among the different training modalities, walking has been widely recommended by several guidelines.^1^^,^^2^^,^^9^ However, the chronic effects of training are thought to result from the sum of acute bout responses,^10^ which reinforces the importance of performing daily walking sessions to optimize chronic adaptations. However, acutely, each walking session may transiently increase cardiovascular risk.^10^ Indeed, previous studies have reported that walking to near-maximal IC symptoms increases cardiac overload, endothelial dysfunction, oxidative stress, and inflammation,^7^^,^^11^^-^^13^ which enhances the risk for ischemia and arrhythmias in predisposed patients.^14^

Accordingly, maximal walking may have hazardous post-exercise effects in patients with symptomatic PAD, and submaximal walking (until moderate leg pain) appears as a potential option that may promote lower post-exercise cardiac overload accompanied by moderate oxidative stress and inflammation. Novakovic et al.^15^ have shown that walking at moderate pain improved several outcomes in these patients, such as vascular function. Additionally, previous studies have tested a specific submaximal walking protocol (15 bouts of 2 minutes of walking at pain threshold) and reported that it induces tolerable levels of leg pain and moderate metabolic and cardiovascular stimuli during its execution,^16^ induces post-exercise hypotension,^17^ and improves walking capacity and cardiovascular parameters after a period of regular training.^8^

Thus, the aim of this study was to compare, in patients with symptomatic PAD, the acute effects of maximal and submaximal walking exercises on the following post-exercise variables: i) cardiovascular function, assessed by BP, heart rate (HR) and rate-pressure product (RPP); ii) cardiac autonomic modulation, assessed by low (LF) and high-frequency (HF) components of HR variability and LF/HF ratio; iii) vascular function, assessed by forearm and calf blood flows (BF) and BF responses to reactive hyperemia; iv) endothelial function, assessed by nitric oxide (NO) bioavailability; v) oxidative stress, assessed by lipid peroxidation; and vi) inflammation, assessed by C-reactive protein (CRP), tumor necrosis factor-α (TNF-α), vascular cell adhesion protein (VCAM), and intercellular adhesion molecule (ICAM). The following were hypothesized: i) a maximal walking session would increase post-exercise cardiac overload (BP, HR, and RPP), sympathovagal balance (LF and LF/HF ratio), and vascular dysfunction, while the submaximal walking session would decrease BP and RPP, while inducing a lower increase on HR and sympathovagal balance; and ii) maximal and submaximal walking sessions would increase post-exercise oxidative stress and inflammation with greater responses after maximal walking.

## Methods

This single center study followed a non-random repeated measurement design in which each patient underwent two experimental sessions conducted in a fixed order. The study protocol followed the Declaration of Helsinki. It was registered at the Brazilian Clinical Trials website (http://www.ensaiosclinicos.gov.br, RBR-3pq58k) and was approved by the Joint Committee on Ethics of Human Research of the School of Physical Education and Sport at the University of São Paulo (process 667.382). Written informed consent was obtained from all participants.

### Participants

Patients were recruited from those assisted at the Vascular Unit of the Hospital das Clínicas of the University of São Paulo, Brazil, according to the possibility of contacting them. Male patients previously diagnosed with PAD and IC were invited. Inclusion criteria were the following: a) age ≥ 50 years; b) ankle-brachial index ≤ 0.90 in at least one leg^1^; c) Fontaine stage II (a and b) of PAD^1^; d) body mass index < 35 kg/m^2^; e) resting systolic BP < 160 mmHg and diastolic BP < 105 mmHg; f) not currently taking β-blockers or non-dihydropyridine calcium channel blockers; g) ability to walk at least 2 minutes at 3.2 km/h on a treadmill; h) ability to undertake an incremental treadmill test limited by symptoms of IC; and i) absence of myocardial ischemia or complex arrhythmias during a treadmill test.

### Preliminary evaluations

All patients underwent preliminary evaluation to identify whether they met the study criteria. They were interviewed to assess the following: age, presence of cardiovascular disease, risk factors, comorbid conditions, and current medication. Ankle-brachial index was measured as previously described.^1^ Body mass and height were assessed with standard equipment (Welmy 110, Brazil), and body mass index was calculated. Resting brachial BP was measured by the auscultatory method after 5 minutes of seated rest. Three measurements were taken in each of 2 visits, and the mean value was calculated for each arm. The highest mean value was also documented. Finally, all patients undertook an exercise test on a treadmill following Gardner's protocol (3.2 km/h with 2% increase in grade per minute)^18^ until maximal claudication pain was experienced. This test was also employed as a familiarization to the maximal effort.

### Experimental protocol

Following the preliminary procedures, patients who fulfilled all study criteria underwent the experimental protocol that consisted of both experimental sessions, maximal and submaximal walking. The submaximal walking session was performed after the maximal session with an interval of at least 7 days between sessions. Moreover, all patients underwent 2 familiarization sessions before undergoing the submaximal session. During each session, cardiovascular, autonomic, endothelial, oxidative stress, and inflammatory variables were evaluated prior to and after the submaximal or maximal walking protocols.

Before both sessions, the patients were instructed to maintain similar routines for the prior 24 hours. In addition, they were instructed to avoid physical exercise for the previous 48 hours, alcoholic beverages for the previous 24 hours, and smoking on the day of the sessions. They were also instructed to take their medication regularly and to attend to the laboratory in a fasted state.

The sessions were conducted in a temperature-controlled laboratory (20 to 22 °C). Patients arrived at 7 am and received a standardized meal (two cereal bars and 50 ml of juice).^19^^,^^20^ A catheter was then inserted into the antecubital vein of the left arm and kept patent by sterile saline. The patients then rested in the supine position for 20 minutes until the commencement of the experimental procedures.

Experimental procedures were initiated at 8 am with pre-exercise assessments performed in the supine position after a 10-minute stabilization period. Electrocardiogram (ECG) and respiration were recorded between 10 and 20 minutes to assess cardiac autonomic modulation. Auscultatory BP and HR were measured in triplicate between 20 and 25 minutes, and the mean value was used for analysis. A venous blood sample was then collected followed by the assessment of lower and upper limb BF and vasodilatory responses to reactive hyperaemia.

Subsequently, patients performed the walking exercise on a treadmill. In the maximal session, they walked at 3.2 km/h with grade increased 2% every minute until maximal pain (Gardner's protocol).^18^ During the submaximal session, they performed 15 bouts of 2 minutes of walking separated by 2 minutes of upright rest, as previously described.^8^^,^^16^^,^^17^ Treadmill speed was maintained at 3.2 km/h with the grade adjusted to maintain the HR of the pain threshold (i.e. the HR measured when the patients had experienced initial claudication pain during the preliminary maximal walking test).

At the end of the walking sessions, the patients immediately returned to supine position for the post-exercise assessments that included an immediate blood sampling. At 20 to 30 minutes of recovery, ECG and breathing movements were recorded for cardiac autonomic modulation assessment, followed by the assessments of auscultatory BP and HR in triplicate. Finally BF and vasodilatory responses were recorded.

### Measurements

#### Cardiovascular function

Recordings of ECG were obtained at D2 (EMG System, Brazil) with HR determined by the ECG. Respiratory signal was obtained by a piezoelectric belt (UFI, Pneumotrace2, USA) positioned at the patients’ thorax. Auscultatory BP was measured in the dominant arm using a mercury sphygmomanometer (Unitec, Brazil), and mean BP was calculated. RPP was calculated by the product of HR and systolic BP as a marker of myocardial oxygen consumption and, thus, of cardiac overload.^21^

#### Cardiac autonomic modulation

For cardiac autonomic evaluation, R-R intervals from the ECG and respiratory signals from the thoracic belt were inputted into a data acquisition system (WinDaq, DI-720, Akron, USA) at a sampling rate of 500 Hz/channel. Stationary segments of 250 to 300 beats were analyzed via spectral analysis of HR variability using the autoregressive method (Heart Scope, version 1.3.0.1, AMPS-LLC, USA). LF (LF_RR_, 0.04 – 0.15 Hz) and HF (HF_RR_, 0.15 – 0.4 Hz) components of HR variability were calculated and expressed in normalized units (nu). The LF/HF ratio was also calculated. All procedures followed the Task Force for HR variability.^22^

#### Vascular function

BF were simultaneously determined in the dominant forearm and the leg with the lowest ankle-brachial index, via venous occlusion plethysmography (Hokanson, AI6, USA).^23^ Briefly, BF to the hand and the foot were interrupted by cuffs inflated to 200 mmHg positioned, respectively, around the wrist and the ankle. Other cuffs placed at the arm and the thigh were rapidly inflated for 10 seconds at 40 to 60 mmHg, followed by 10 seconds of deflation. Increases in forearm and calf volumes were detected by mercury strain gauges positioned at the largest circumference of these limb segments and recorded by specialized software (NIVP3; Hokanson, USA). Measurements were taken for 4 minutes (twelve 20-second cycles) and the first 2 and the last cycle measurement were excluded from analysis (i.e. mean of 9 cycles). Forearm and calf vasodilatory responses to reactive hyperemia were assessed immediately after determination of BF.^23^ For this, BF to each limb was occluded for 5 minutes by inflating the thigh and forearm cuffs to 200 mmHg. Afterwards, the cuffs were released and post-occlusion BF were measured for 4 minutes as previously described. Vasodilatory response was calculated as the difference in the area under the curve (AUCBF) of the post- and pre-hyperemia BF measurements.

#### Blood analysis

In each sampling moment, 15 ml of blood were collected in standard anticoagulant EDTA-treated vacutainer tubes. Samples were centrifuged within 30 minutes, divided into aliquots and stored at –80 °C until analysis. Plasma concentrations of CRP, TNF-α, VCAM, and ICAM were determined by enzyme-linked immune-sorbent assays (ELISA) according to the manufacturer's instructions in each kit (Cayman Chemical, USA for CRP; and R&D Systems, USA for TNF-α, VCAM, and ICAM). Lipid peroxidation was analyzed by specific kits (Cayman Chemical, USA), and NO was analyzed by the chemiluminescence method with a specific analyzer (Sievers ® Nitric Oxide Analyzer NOA 280, USA).

### Statistical analyses

Considering a power of 90%, an alpha error of 5%, and a standard deviation of 3 mmHg for systolic BP and 0.6 ml.100 ml tissue^−1^.min^−1^ for BF (i.e. the main clinical outcomes), the minimal sample sizes necessary to detect a difference of 4 mmHg in systolic BP and 0.5 ml.100 ml tissue^−1^.min^−1^ in BF were calculated to be 10 and 14 subjects, respectively. As other variables with greater variation were included in the study, the sample size used was greater.

Normality and homogeneity of variance for all data were checked using the Shapiro-Wilk and Levene tests, respectively. When non-normality of data was identified, a logarithmic transformation was applied, and normal distribution was obtained. Responses to walking sessions were compared by two-way ANOVA (Statsoft, Statistic for Windows 4.3, Oklahoma, USA) for repeated measures with session (maximal versus submaximal) and moment (pre- versus post-exercise) as the main factors. When pre-exercise values were significantly different between the sessions (i.e. for HR and RPP), an analysis of covariance (ANCOVA) was employed using the pre-exercise value as a covariate. The Newman-Keuls post-hoc test was used to identify significances when appropriate. P < 0.05 was considered significant, and data were presented as mean ± standard deviation for continuous variables and as frequency of appearance (%) for categorical variables, such as comorbidities and medication use.

## Results

Fifty patients volunteered for the study, and 11 refrained from participating due to lack of time. Thus, 39 patients signed the informed consent and performed the preliminary examinations, 9 of which were excluded (5 due to ECG abnormalities in the exercise test and 4 due to interruption of exercise test for reasons other than claudication pain). Therefore, 30 patients underwent both the maximal and submaximal experimental sessions and their characteristics are shown in Table 1.

**Table 1:** Patient characteristics

	Mean ± standard deviation
Age (years)	66 ± 11
Body mass index (kg/m^2^)	25.3 ± 3.2
**Diagnosis of PAD**	
ABI at rest	0.62 ± 0.12
COD (m)	218 ± 87
TWD (m)	606 ± 275
**Comorbidities**	
Obesity (%)	10.0
Hypertension (%)	73.3
Diabetes mellitus (%)	26.7
Dyslipidemia (%)	93.3
Current smokers (%)	33.3
Heart disease/stroke (%)	23.3
**Drug therapy**	
Aspirin (%)	93.3
Statin (%)	93.3
Antihypertensive agent (%)	60.0
Oral hypoglycemic (%)	26.7

Data are mean ± standard deviation or percentage (%). ABI: ankle-brachial index; COD: claudication onset distance; PAD: peripheral artery disease; TWD: total walking distance. Obesity defined as body mass index ≥ 30 kg/m^2^. Diabetes, hypertension, dyslipidemia, heart disease, and stroke defined by previous medical diagnosis.

Hemodynamic and autonomic responses are shown in Table 2. Systolic and mean BP decreased after the submaximal session and increased after the maximal session (interactions, p < 0.001 for both). Diastolic BP increased only after maximal walking (interaction, p < 0.001). Pre-exercise HR and RPP were significantly higher in the submaximal session than the maximal walking one, and ANCOVA revealed that these pre-exercise differences did not affect the results. Thus, HR displayed similar increases after both the maximal and the submaximal walking bouts (moment, p < 0.001), while RPP increased significantly only after maximal walking (interaction, p = 0.007).

**Table 2:** Hemodynamic and autonomic variables measured pre- and post-exercise in the submaximal and maximal walking sessions

	Submaximal		Maximal				
	Pre	Post	Pre	Post	p session	p moment	p interaction
**Systemic hemodynamics (N = 30)**							
Systolic BP (mmHg)	132 ± 16	125 ± 15*#	134 ± 13	138 ± 17*	0.01	0.18	0.01
Diastolic BP (mmHg)	77 ± 9	76 ± 8#	78 ± 8	83 ± 9*	0.01	0.01	0.01
Mean BP (mmHg)	95 ± 10	92 ± 9*#	96 ± 9	101 ± 10*	0.01	0.05	0.01
HR (bpm)	64 ± 9#	67 ± 9*#	68 ± 9	71 ± 10*	0.01	0.01	0.70
RPP (bpm* mmHg)	8466 ± 1466#	8308 ± 1433#	9010 ± 1394	9762 ± 1671*	0.01	0.01	0.01
**Autonomic modulation (n=22)**							
LF (nu)	56 ± 22	64 ± 20*	51 ± 18	60 ± 21*	0.12	0.05	0.75
HF (nu)	40 ± 21	31 ± 19*	44 ± 17	34 ± 19*	0.19	0.02	0.88
LF/HF ratio	0.2 ± 0.5	0.4 ± 0.4*	0.1 ± 0.4	0.3 ± 0.5*	0.11	0.04	0.74
**Local hemodynamics (n = 21)**							
Forearm BF	1.42 ± 0.63	1.68 ± 0.68*	1.41 ± 0.59	1.65 ± 0.67*	0.70	0.01	0.59
Calf BF	12.0 ± 7.1	13.7 ± 7.0*	12.2 ± 7.8	14.4 ± 8.8*	0.11	0.01	0.57
Forearm VR	80.9 ± 34.8	67.2 ± 30.0*	83.9 ± 40.6	73.6 ± 34.1*	0.18	0.01	0.14
Calf VR	56.8 ± 30.2	40.4 ± 19.8*	63.9 ± 29.5	52.4 ± 26.5*	0.02	0.01	0.52
Forearm AUCBF	1085 ± 507	1299 ± 609	1294 ± 676	1218 ± 476	0.66	0.38	0.50
Calf AUCBF	1081 ± 606	1152 ± 603	999 ± 467	1270 ± 825	0.89	0.13	0.20

Data are mean ± standard deviation. AUC: area under the curve; BF: blood flow; BP: blood pressure; HF: high frequency; HR: heart rate; LF: low frequency; nu: normalized units; RPP: rate-pressure product; VR: vascular resistance. Values for BF are ml.100 ml tissue-1.min-1.

* different from pre in the same session (p < 0.05)

# different from the maximal session at the same moment (p < 0.05). Analyses performed by two-way ANOVA.

HF decreased while LF and the LF/HF ratio increased significantly and similarly after both the maximal and the submaximal walking sessions (moment, p = 0.02, p = 0.05, and p = 0.04, respectively).

Forearm and calf BF increased significantly and similarly after the maximal and the submaximal walking bouts (moment, p < 0.001), while forearm and calf vascular resistance decreased similarly after both walking bouts (moment, p < 0.001 and p = 0.01, respectively), and forearm and calf AUCBF did not change after either submaximal or maximal walking bouts (all p > 0.05).

Blood responses are shown in Table 3. NO, lipid peroxidation, CRP, and TNF-α did not change after either submaximal or maximal walking bouts (all p > 0.05), while ICAM and VCAM displayed a similar and significant increase after the maximal and the submaximal walking sessions (moment, p = 0.001 for both).

**Table 3:** Plasma concentrations of nitric oxide, oxidative stress, and inflammatory variables measured pre- and post-exercise in the submaximal and the maximal walking sessions

	Submaximal		Maximal				
	Pre	Post	Pre	Post	p session	p moment	p interaction
NO (μM)	14.32±5.65	13.59±4.63	13.53±4.51	13.68±4.21	0.29	0.24	0.57
**Oxidative stress**							
LPO (μM)	18.81±14.69	19.29±15.34	18.71±17.06	20.55±19.01	0.81	0.44	0.77
**Inflammation**							
CRP (pg/ml)	1868±1435	1843±1485	1614±1651	1837±1586	0.41	0.13	0.45
TNF-α (pg/ml)	1.18±0.36	1.24±0.29	1.21±0.28	1.23±0.25	0.75	0.21	0.57
ICAM (ng/ml)	223±96	236±99*	218±92	244±100*	0.74	0.01	0.08
VCAM (ng/ml)	619±250	671±286*	592±237	650±247*	0.16	0.01	0.75

Data are mean ± standard deviation. CRP: C-reactive protein; ICAM: intercellular adhesion molecule; LPO: lipid peroxidation; NO: nitric oxide; TNF-α: tumor necrosis factor-α; VCAM: vascular cell adhesion protein.

* different from pre in the same session (p < 0.05). Analyses performed by two-way ANOVA.

## Discussion

The main findings of this study were that patients with symptomatic PAD presented the following: 1) a reduction in systolic BP after submaximal walking, as well as an increase in systolic BP after maximal walking; 2) an increase in RPP only after the maximal walking; 3) similar increases in HR, LF/HF ratio, LF, ICAM, and VCAM levels after the maximal and submaximal walking sessions; and 4) no changes in NO and vasodilatory capacity after either maximal or submaximal walking sessions.

Walking to submaximal, but not maximal pain decreased post-exercise BP. Previous studies^17^^,^^24^ have already reported the occurrence of post-exercise hypotension (PEH, i.e., a decrease in BP after an exercise bout in comparison to pre-exercise values)^25^^,^^26^ in patients with symptomatic PAD after walking to moderate pain. The novelty of this study was to provide evidence that, in patients with PAD at Fontaine stage II, PEH did not occur when walking was performed to maximal pain, and BP remained elevated after maximal walking. As PEH is known as a clinically relevant phenomenon in hypertensive populations,^27^ submaximal, but not maximal walking may produce acute hypotensive benefits in patients with IC and hypertension. Moreover, recent evidence has shown that PEH correlates with decreases in BP after a training period, and it is a possible predictor of the chronic responsiveness.^28^^,^^29^ Thus, these results raise the hypothesis that submaximal walking might produce better chronic hypotensive effects than maximal walking in this population. This needs to be tested by future studies.

Post-exercise HR increased similarly after the maximal and submaximal walking sessions, which is consistent with the similar increase observed in cardiac autonomic modulation changes towards sympathetic predominance after both walking sessions (i.e. a similar increase in LF and the LF/HF ratio, as well as a decrease in HF).^30^ This lack of difference between the maximal and submaximal sessions was, to a certain extent, unexpected, given that, in other populations, changes in post-exercise HR and sympathovagal modulation are usually associated with exercise intensity.^31^ This apparently contradictory result may be explained by the fact that the submaximal walking session lasted longer (30 minutes, total distance walked = 1600 m) than the maximal session (12 ± 5 minutes, total distance walked = 606 ± 275 m). Thus, as pain produces sympathetic activation,^32^ it is possible that, despite the moderate intensity, the longer period of pain in the submaximal session may have led to a sustained increase in sympathetic modulation and, consequently, HR during the recovery period, matching the increase produced by the more intense but shorter maximal session. Additionally, although post-exercise HR increased in both walking sessions, BP decreased only in the submaximal walking session, consequently leading to higher RPP after maximal walking, which reflects greater cardiac overload, and, consequently, greater risk of acute adverse events after maximal walking.^14^ Thus, these results suggest that submaximal walking may be safer for patients predisposed to acute cardiovascular events.

Forearm and calf BF increased similarly after the submaximal and maximal walking sessions, and these responses are in agreement with previous studies.^17^^,^^33^ However, interestingly, vasodilatory capacity did not change after either walking session, whereas previous studies reported decreased endothelial function after maximal walking.^12^^,^^34^ Possible differences among the studies may be related to the methods used to assess vascular function (plethysmograph versus ultrasound). Nevertheless, in the current study, the absence of change in vasodilatory capacity is in accordance with the maintenance of NO and oxidative stress markers.

As expected, maximal and submaximal walking sessions increased inflammatory markers. However, different from the hypothesis, inflammation increased similarly after both sessions. Once again, this response may be related to the fact that exercise duration was longer in the submaximal walking session, leading to similar magnitude of inflammation, in spite of lower pain.

The absence of paired volume between the two walking sessions is a limitation to this study, which precludes us from attributing the results solely to the degree of pain. However, as a first study comparing post-exercise maximal and submaximal responses, this study opted to use a maximal protocol extensively investigated in literature^7^^,^^11^^,^^34^ and a submaximal protocol, both of which have already been demonstrated to elicit cardiovascular benefits.^8^^,^^17^ Future studies should compare other maximal and submaximal protocols with similar volume. Additionally, it is important to mention that this study was conducted with men at Fountain stage IIa and IIb, and post-walking responses may differ in women, in patients at other stages of the disease, and in patients with different clinical characteristics, notwithstanding Fontaine stage II. Future studies can overcome these limitations by studying women and other patients with PAD. In addition, measurements were performed only in one time-point during the post-exercise period. For a better understanding of responses, a follow-up for a longer period, with more measurements, should be performed in future investigations.

## Conclusions

In male patients with symptomatic PAD, walking to submaximal, but not maximal pain reduces post-exercise BP, while only maximal walking elevates post-exercise RPP. On the other hand, maximal and submaximal walking sessions produce similar post-exercise increases in HR, cardiac sympathovagal balance, BF, and inflammation.

### Practical implications

- Submaximal, but not maximal walking reduces BP in the post-exercise period.
- Only maximal walking increases post-exercise cardiac load.
- Submaximal and maximal walking similarly increase post-exercise inflammation.
- Submaximal walking might be more adequate than maximal walking for patients with symptomatic PAD, because it results in lower acute cardiovascular risk during the recovery period.
